# Electronic and Magnetic Properties of Defected Monolayer WSe_2_ with Vacancies

**DOI:** 10.1186/s11671-019-3002-2

**Published:** 2019-06-04

**Authors:** Danxi Yang, Xiaoli Fan, Fengxia Zhang, Yan Hu, Zhifen Luo

**Affiliations:** State Key Laboratory of Solidification Processing, Centre of Advanced Lubrication and Seal Materials, School of Material Science and Engineering, Northwestern Polytechnical University, 127 YouYi Western Road, Xi’an, 710072 Shannxi China

**Keywords:** Monolayer WSe_2_, Vacancy, External strain, Electronic properties, Magnetic properties, First-principle calculations

## Abstract

By adopting the first-principle methods based on the density functional theory, we studied the structural, electronic, and magnetic properties of defected monolayer WSe_2_ with vacancies and the influences of external strain on the defected configurations. Our calculations show that the two W atom vacancies (V_W2_) and one W atom and its nearby three pairs of Se atom vacancies (V_WSe6_) both induce magnetism into monolayer WSe_2_ with magnetic moments of 2 and 6 μ_B_, respectively. The magnetic moments are mainly contributed by the atoms around the vacancies. Particularly, monolayer WSe_2_ with V_W2_ is half-metallic. Additionally, one Se and one W atom vacancies (V_Se_, V_W_), two Se atom vacancies (V_Se-Se_), and one W atom and the nearby three Se atoms on the same layer vacancy (V_WSe3_)-doped monolayer WSe_2_ remain as non-magnetic semiconducting. But the impure electronic states attributed from the W d and Se p orbitals around the vacancies locate around the Fermi level and narrow down the energy gaps. Meanwhile, our calculations indicate that the tensile strain of 0~7% not only manipulates the electronic properties of defected monolayer WSe_2_ with vacancies by narrowing down their energy gaps, but also controls the magnetic moments of V_W_-, V_W2_-, and V_WSe6_-doped monolayer WSe_2_.

## Introduction

Unlike gapless graphene [1, 2], semiconducting transition metal dichalcogenide (TMD) monolayers with a band gap of 1~2 eV [3–6] have superior advantages in the fields of catalyst, electronics, and optoelectronics because of their unique chemical, optical, and electronic properties [3–9]. Particularly, monolayer WSe_2_ is semiconducting with a direct band gap of ~ 1.6 eV [4, 10–12]. Additionally, its carrier mobility is around 250 cm^2^/V, and the on/off ratio is higher than 10^6^ at room temperature [13]. More importantly, monolayer WSe_2_ is the first TMD showing p-type conducting behavior with high work function metal (Pd) being the contacts [13]. Because of these novel properties, monolayer WSe_2_ has been widely studied as the promising candidate in the future electronics and optoelectronics [4, 6, 13–16]. However, monolayer WSe_2_ is non-magnetic which limits its application in many other fields related with magnetism.

Based on the previous studies [17–25], structural defects significantly influence the mechanical, electronic, and magnetic properties. For example, point defect and vacancy defect introduce magnetism into graphene [19, 20], MoS_2_ monolayer, and BaTiO_3_(001) thin film [21–23], respectively. Wu et al. studied the effects of defects on the device transmission performance in monolayer WSe_2_ tunneling field-effect transistors (TFSTs) by performing the ab initio calculation, which indicates that defects can be well designed to obtain high-performance TFETs [25]. Meanwhile, structural defects were found in the as-grown 2D materials due to the imperfection of the growth process [19, 20, 26–28]. For example, intrinsic structural defects, such as point defects, are noticeable in the as-grown monolayer WSe_2_ [26].

Indeed, structural engineering methods including irradiation by high energy particles of electron beam [29], ion beam [30] and high energy laser, and chemical etching [31, 32] are the effective techniques to induce defects in the 2D materials and have been used to modify the atomic structures. Therefore, it is not only significant but also realistic to study the influence of structural defects such as vacancies on the properties of monolayer WSe_2_, which may offer us the new feature. Additionally, the 2D materials can withstand large strains before rupture and even be stretched beyond the inherent limit of 10% owing to their strong plastic deformation ability as demonstrated on monolayer MoS_2_ [33, 34]. Thus, strain engineering has been widely used to tune the properties of 2D materials and enhance the relevant performance in the related applications [11, 17, 33–39]. According to Yang et al.’s study, nanoscale local strain modifies the optical band gap and changes the electronic and magnetic properties of monolayer ReSe_2_ [38]. Particularly, it was reported that the non-magnetic WS_2_ monolayer becomes ferromagnetic under the applied biaxial strain, and the highest magnetic moment reaches 4.85 μ_B_ [39].

In this work, we systematically investigated the effects of vacancy defects and tensile strain on the electronic properties of monolayer WSe_2_. We calculated several vacancy defects of single atom vacancy, double atom vacancy, and big vacancies of four and seven atoms. We found that all the vacancy defects change the electronic properties of monolayer WSe_2_, while only the V_W2_ and V_WSe6_ vacancies introduce the magnetism of 2 and 6 μ_B_, respectively. Additionally, monolayer WSe_2_ with V_W_ vacancy converts into magnetic from non-magnetic under the external tensile strain. More importantly, the external biaxial strain effectively modulates not only the energy gaps but also the magnetic moments of V_W_-, V_W2_-, and V_WSe6_-doped monolayer WSe_2_. Our calculations suggest defected monolayer WSe_2_ with vacancies as potential monolayer magnetic semiconductors.

## Computational Methods

All the calculations in the present study were performed by adopting the Vienna Ab initio Simulation Package (VASP) based on density functional theory (DFT) [40, 41]. The Perdew–Burke–Ernzerhof (PBE) method was used to calculate the electronic exchange interaction [42]. The ion–electron and electron–electron interactions were calculated by the projector augmented wave (PAW) method and the plane wave basis set [43, 44]. The cutoff energy for the plane wave basis set was set to 300 eV, and the first Brillouin zone was sampled by the 3 × 3× 1 k-mesh based on the Monkhorst–Pack method [45]. A vacuum space of 15 Å was added along the vertical direction above the monolayer to remove the interactions between the adjacent images in the periodic slab model. Structure relaxations have been carried out until all the forces on each ion are less than 0.02 eV/Å, and the convergence criteria for the total energy were set as 10^−4^ eV. The biaxial tensile strain was imposed on the vacancy defect–doped monolayer WSe_2_, which was calculated by *ε* = (*c* − *c*_0_)/*c*_0_ × 100%, where *c* and *c*_0_ are the lattice parameters of the strained and free monolayer WSe_2_, respectively.

## Results and Discussion

### Atomic Structure and Electronic Properties of Monolayer WSe_2_

**Fig. 1:**
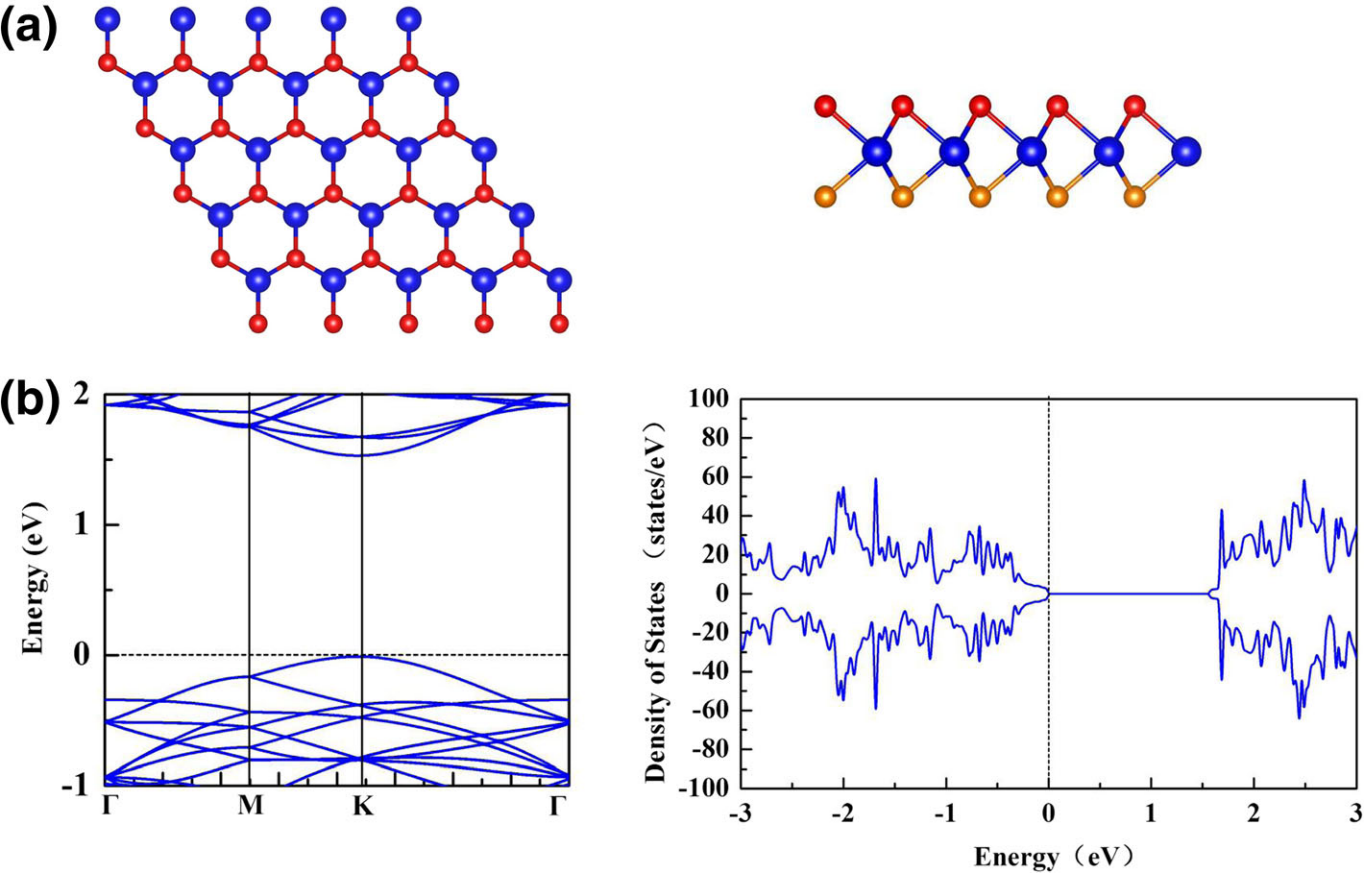
**a** Top and side views for the atomic structure of monolayer WSe_2_. **b** The electronic band structure and density of states (DOS) of monolayer WSe_2_. The blue, red, and tangerine balls represent Wand Se atoms on the top and bottom layer, respectively. Fermi level is set as 0 eV

The most stable crystal structure of monolayer WSe_2_, denoted as 1H-WSe_2_, is shown in Fig. 1a, which shows the sandwiched layer of Se-WSe. In 1H-WSe_2_, W atoms and Se atoms occupy the sublattices of hexagonal sheet, and the Se atoms on the lower layer are directly underneath those Se atoms on the upper layer. Our calculated W-W bond length is 3.31 Å and the W-Se bond length is 2.54 Å, agreeing well with previous results [10, 11]. As shown in Fig. 1b, the calculated electronic band structure and density of states (DOS) for 1H-WSe_2_ indicate that 1H-WSe_2_ is non-magnetic semiconducting with a direct band gap of 1.54 eV. Our calculated result agrees well with the previous result of 1.55 eV [12]. To get a more accurate band gap, we adopted the Heyd–Scuseria–Ernzerh (HSE06) [46] method to calculate the electronic band structure. The energy gap of 1H-WSe_2_ calculated by HSE06 method is 2.0 eV.

### The Magnetic and Electronic Properties of Defected Monolayer WSe_2_ with Vacancy

**Fig. 2:**
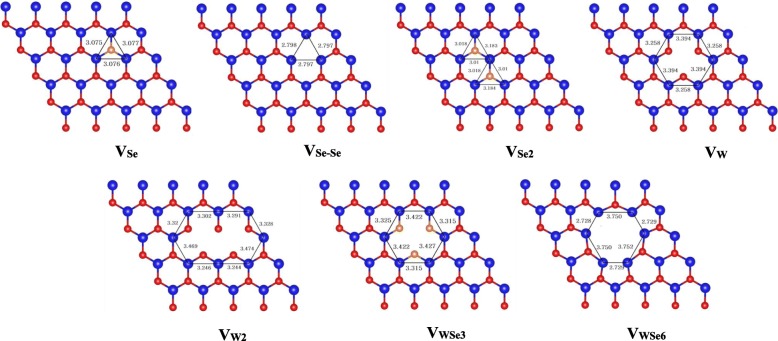
The optimized atomic structures of monolayer WSe_2_ with V_Se_, V_Se-Se_, V_Se2_, V_W_, V_W2_, V_WSe3_, and V_WSe6_ vacancies. The blue, red, and tangerine balls represent W and Se atoms on the top and bottom layer, respectively

We considered seven vacancy defect configurations for monolayer WSe_2_ in the present study. They are the single atom vacancies including one Se atom vacancy (V_Se_), one W atom vacancy (V_W_), and two atom vacancies of V_Se-Se_, V_Se2_, and V_W2_. The two Se atom vacancy V_Se-Se_ means the two Se atoms which are just beneath or above each other are removed, while the V_Se2_/V_W2_ vacancy means that the two adjacent Se/W atoms are removed. We also considered the big vacancies of V_WSe3_ and V_WSe6_. V_WSe3_ denotes the vacancy of one W atom and the nearby three Se atoms on the same layer, and V_WSe6_ presents the vacancy of one W atom and the nearby three pairs of Se atoms. The optimized structures of monolayer WSe_2_ with vacancies of V_Se_, V_Se-Se_, V_Se2_, V_W_, V_W2_, V_WSe3_, and V_WSe6_ are shown in the insets of Fig. 2. As we can see, the 5 × 5 × 1 supercell was used for the present study of the defected monolayer WSe_2_.

Table 1 summarizes the results for the defected monolayer WSe_2_ with vacancies of V_Se_, V_Se-Se_, V_Se2_, V_W_, V_W2_, V_WSe3_, and V_WSe6_. We can see that the W-W distances around the vacancies of V_Se_, V_Se-Se_, and V_Se2_ decrease respectively by 0.23, 0.52, and 0.24 Å compared with the original W-W distance in monolayer WSe_2_, which means that the W atoms around the Se atoms vacancies get close to each other. Additionally, the W-W distances around the vacancies of V_W_, V_W2_, and V_WSe3_ slightly increase by 0.02, 0.01, and 0.06 Å. And those W-W distances around the single atom vacancies (V_Se_ /V_W_) are almost equal to the counterpart around the two atoms vacancies (V_Se2_/V_W2_). For the bigger vacancy V_WSe6_-doped monolayer WSe_2_, the W-W distances between the neighboring W atoms at the corners of the vacancy reduce by 0.58 Å, but the W-W distances at the edges of the vacancy increase by 0.44 Å. The formation energies of the seven vacancy geometries are calculated via:

$$ {E}_{\mathrm{form}}={E}_{\mathrm{van}\hbox{-} {\mathrm{WSe}}_2}\hbox{-} {E}_{{\mathrm{WSe}}_2}+\Sigma {n}_{\mathrm{i}}{u}_{\mathrm{i}} $$

**Table 1:** The calculation results for monolayer WSe_2_ with V_Se_, V_Se-Se_, V_Se2_, V_W_, V_W2_, V_WSe3,_ and V_WSe6_ vacancies

	1H-WSe_2_	V_Se_	V_Se ‐ Se_	V_Se2_	V_W_	V_W2_	V_WSe3_	V_WSe6_
d_W ‐ W_ (Å)	3.31	3.08	2.79	3.07	3.33	3.32	3.37	2.73^a^/3.75^b^
E_gap_ (eV)	1.54	1.18	1.15	1.02	0.18	0.19 ^c^	0.76	0.1
M_tot_ ( μ_B_)	0	0	0	0	0	2	0	6
E_form_ (eV)	–	2.66	4.7	5.39	5.35	9.43	8.85	16.55

*d*_*W* ‐ *W*_ the averaged W-W distances around the vacancy; *E*_*gap*_ and *M*_*tot*_ the energy gaps and total magnetic moments, respectively; *E*_*form*_ the formation energy. The calculation results for the perfect 1H-WSe_2_ are also listed.

^a, b^The W-W distance between the neighboring W atoms at the corners and at the edges around the V_WSe6_, respectively

^c^The energy gap for the half-metal

${E}_{\mathrm{van}\hbox{-} {\mathrm{WSe}}_2}$and ${E}_{{\mathrm{WSe}}_2}$are the total energies of the 5 × 5 × 1 supercell of monolayer WSe_2_ with and without vacancy defect, and *u*_i_ and *n*_i_ (i = Se, W) are the chemical potential and number of the removed *i* atom. As listed in Table 1, our calculated formation energies for the seven vacancies indicate that V_Se_, the single Se atom vacancy, should be frequently observed on WSe_2_ monolayer, consistent with the previous result of monolayer MoS_2_ [17, 21]. For the two Se atom vacancies of V_Se-Se_ and V_Se2_, the formation energy of V_Se2_ is a little higher than that of V_Se-Se_, indicating that V_Se-Se_ is energetically preferable than V_Se2_. Hence, in the following study, only V_Se-Se_ is studied as the two Se atom vacancies. Additionally, the formation energies for the big size vacancies are higher, which may be generated via certain kind of structural engineering techniques [29–31].

**Fig. 3:**
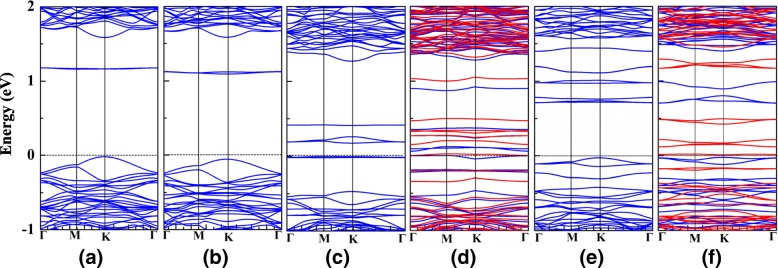
The electronic band structures of monolayer WSe_2_ with **a** V_Se_, **b** V_Se-Se_, **c** V_W_, **d** V_W2_, **e** V_WSe3_, and **f** V_WSe6_ vacancies. Blue and red lines represent the majority and minority spin channels, respectively. Fermi level is set as 0 eV

We then studied the electronic properties of the defected monolayer WSe_2_ with vacancies of V_Se_, V_Se-Se_, V_W_, V_W2_, V_WSe3_, and V_WSe6_. Figure 3 shows the electronic band structures of the six vacancy-doped monolayer WSe_2_. As shown in Fig. 3a, V_Se_-doped monolayer WSe_2_ remains to be semiconducting, but there are obviously extra electronic states generated from the vacancy defect locating in the gap region. Consequently, the energy gap of V_Se_-doped monolayer WSe_2_ reduces to 1.18 eV compared with that of monolayer WSe_2_. The electronic band structure of V_Se-Se_-doped monolayer WSe_2_ is similar with that of V_Se_-doped monolayer WSe_2_, and their energy gaps are close. V_W_- and V_WSe3_-doped monolayer WSe_2_ shown in Fig. 3c and e also maintains the semiconducting feature but with much smaller energy gaps of 0.18 and 0.76 eV, respectively. Different from the above vacancy defects, the majority and the minority spin channels are distributed asymmetrically for the V_W2_- and V_WSe6_-doped monolayer WSe_2_ as shown, in Fig. 3d and f. For the V_W2_-doped monolayer WSe_2_, the majority spin channels cross the Fermi level, while the minority spin channels maintain semiconducting with an energy gap of 0.19 eV, and its magnetic moment is 2.0 μ_B_, while the V_WSe6_-doped monolayer WSe_2_ is magnetic semiconducting with a magnetic moment of 6.0 μ_B_.

**Fig. 4:**
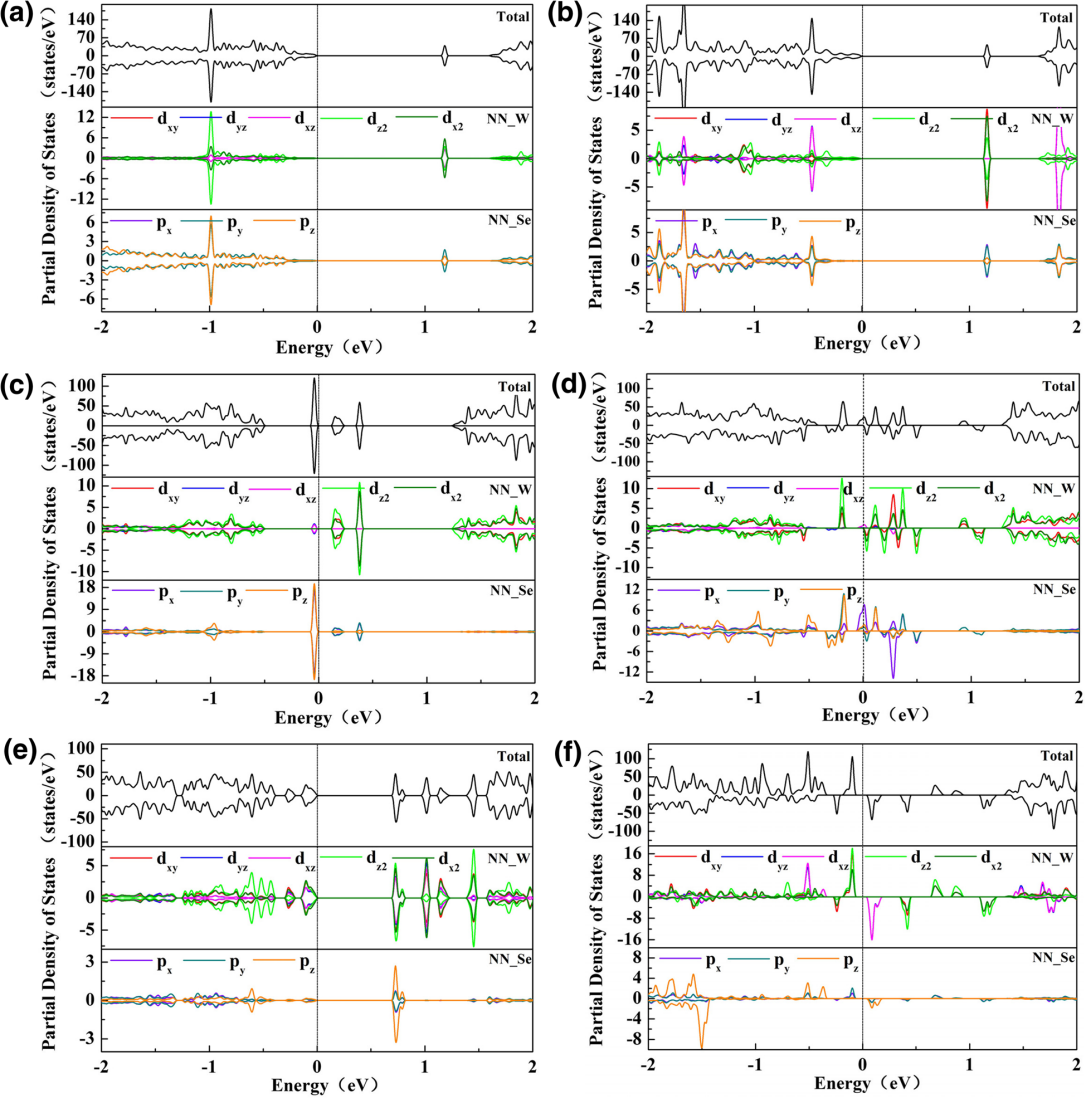
The partial density of states (PDOS) of monolayer WSe_2_ with **a** V_Se_, **b** V_Se-Se_, **c** V_W_, **d** V_W2_, **e** V_WSe3_, and **f** V_WSe6_ vacancies. NN_W and NN_Se represent the nearest neighboring W and Se atoms around the vacancy, respectively. Fermi level is set as 0 eV

We also calculated the partial density of states (PDOS) for the six vacancy-doped monolayer WSe_2_ to further study their electronic properties. Figure 4 shows that the impure electronic states of V_Se_- and V_Se-Se_-doped monolayer WSe_2_ are mostly located in conduction band region, and they are mainly derived from the d orbital of W atoms near the vacancy, and little from p orbital of Se atoms around the vacancy. Differently, the impure electronic bands of V_W_- and V_WSe3_-doped monolayer WSe_2_ are not only located in the conduction band region, but also being split in the valence band region. For V_W_ vacancy, the conduction bands near the Fermi level mainly come from the d (d_xy_, d_x2_and d_z2_) orbitals of the W atoms around the vacancy, and the valence bands near the Fermi level are mainly from the p orbital of Se atoms around the vacancy. Compared with V_W_-doped monolayer WSe_2_, the impure electronic states of V_WSe3_-doped monolayer WSe_2_ are further away from the Fermi level. The conduction bands near the Fermi level are derived from both the Se p_z_orbital and W d orbitals around the vacancy, while the valence bands near the Fermi level are mainly from the W d orbital around the vacancy. Additionally, W d orbital and the neighboring Se p orbital strongly interact, resulting in the hybridized states around the Fermi level. For the half-metallic V_W2_-doped monolayer WSe_2_, the conduction band cross of the Fermi level mainly comes from the Se p_x_orbital, and the valence bands near the Fermi level are mainly derived from the W d (d_x2_ and d_z2_) orbital. As for the magnetic semiconducting V_WSe6_-doped monolayer WSe_2_, the conduction bands and the valence bands near the Fermi level are both derived from the W d orbital near the vacancy.

### The Electronic and Magnetic Properties of Monolayer WSe_2_ with Vacancy Defect Under Tensile Strain

We further studied the electronic and magnetic properties of the vacancy-doped monolayer WSe_2_ under the biaxial strain since the strain is an effective way to tune the electronic structures and magnetic moments of the 2D materials. We firstly studied the 1H-WSe_2_ monolayer under the biaxial strain. Our calculation result shows that the biaxial strain ranging from 0 to 7% does not induce any magnetism into monolayer WSe_2_, similar with monolayer MoS_2_ [34, 36]. Additionally, monolayer WSe_2_ still keeps the semiconducting nature with the energy gap decreasing to 0.5 eV at 7% strain, and the W-W bond length increases as the applied tensile strain increases.

**Fig. 5:**
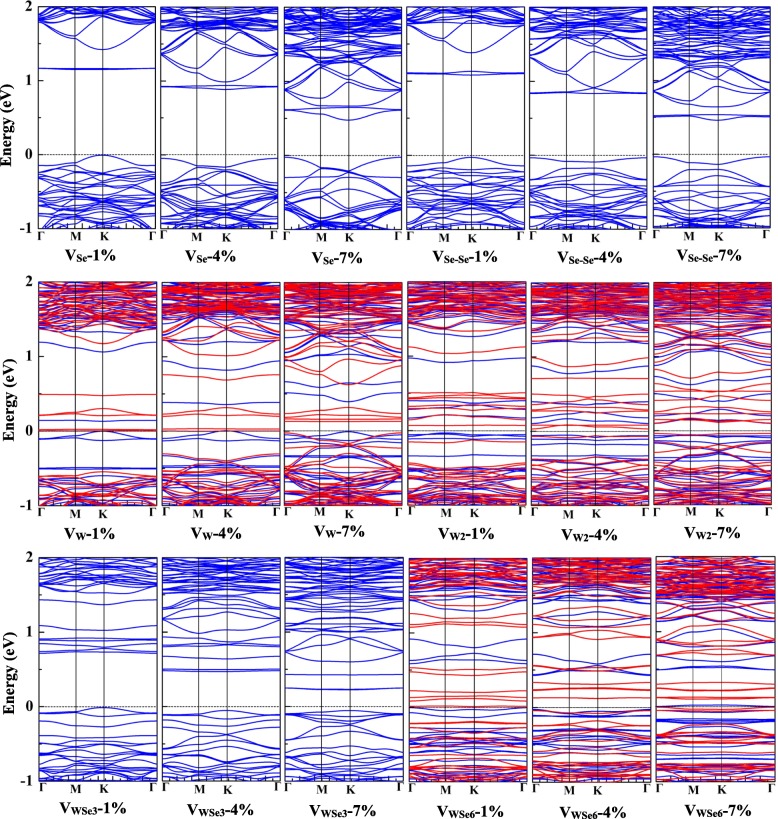
The electronic band structures of monolayer WSe_2_ with V_Se_, V_Se-Se_, V_W_, V_W2_, V_WSe3_, and V_WSe6_ vacancies under 1%, 4%, and 7% tensile strain. Blue and red lines represent the majority and minority spin channels, respectively. Fermi level is set as 0 eV

**Fig. 6:**
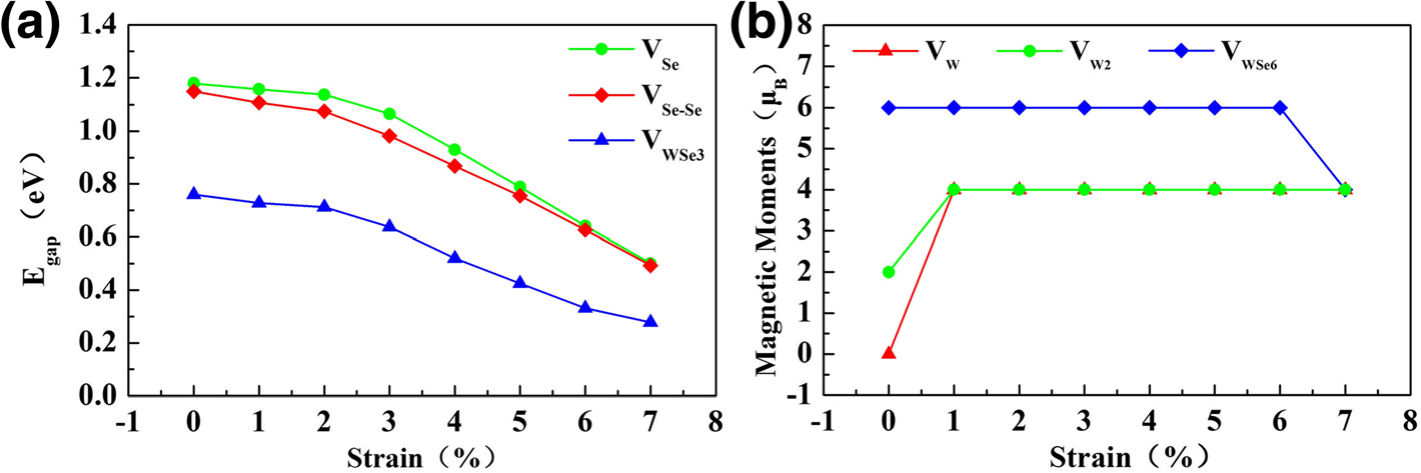
**a** The energy gaps of monolayer WSe_2_ with V_Se_, V_Se-Se_, and V_WSe3_ vacancies. **b** The magnetic moments of monolayer WSe_2_ with V_W_, V_W2_, and V_WSe6_ vacancies under the tensile strain of 0~7%.

Then, we studied the vacancy-doped monolayer WSe_2_ under the tensile strain of 0~7%. Figure 5 shows the electronic band structures for V_Se_-, V_Se-Se_-, V_W_-, V_W2_-, V_WSe3_-, and V_WSe6_-doped monolayer WSe_2_ under the biaxial strain of 1%, 4%, and 7%. Similar with the pristine WSe_2_ monolayer, V_Se_-, V_Se-Se_-, and V_WSe3_-doped monolayer WSe_2_ all maintain the semiconducting feature under the biaxial strain of 0~7%, and the conduction band minima are getting closer to the Fermi level as the applied tensile strain increases. For the V_W_-doped monolayer WSe_2_ under the biaxial strain larger than 1%, the majority and minority spin channels distribute asymmetrically. Additionally, the V_W2_- and V_WSe6_-doped monolayer WSe_2_ both show magnetic semiconducting feature under the strain of 1~7%. Though the V_Se_-, V_Se-Se_-, and V_WSe3_-doped monolayer WSe_2_ still keep the semiconducting feature under the biaxial strain of 0~7%, the biaxial strain effectively controls their energy gaps as shown in Fig. 6a. The energy gaps of V_Se_- and V_Se-Se_-doped monolayer WSe_2_ both decrease from 1.1 to 0.5 eV, while the energy gap of V_WSe3_-doped monolayer WSe_2_ is relatively smaller, which decreased from 0.76 to 0.3 eV. On the other hand, the energy gaps of V_W_-, V_W2_-, and V_WSe6_-doped monolayer WSe_2_ are less than 0.2 eV under the biaxial strain of 0~7%.

**Fig. 7:**
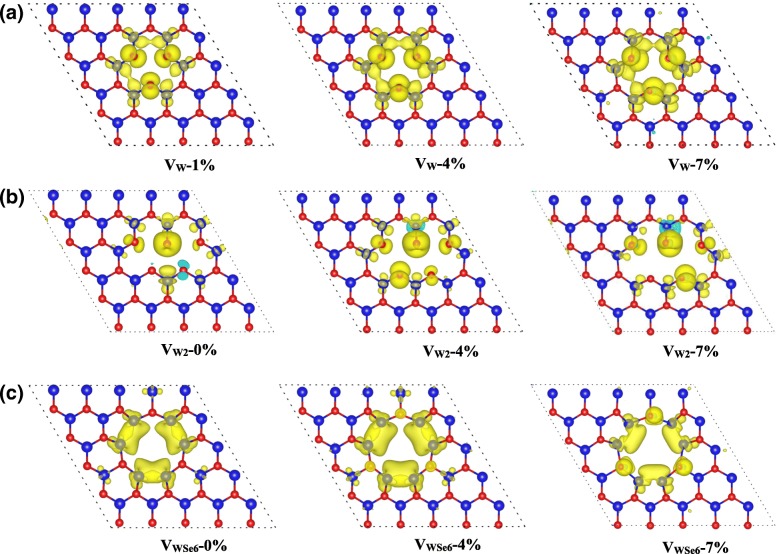
Spin-resolved charge density of monolayer WSe_2_ with **a** V_W_, **b** V_W2_, and **c** V_WSe6_ vacancies under the tensile strain of 0~7%. Yellow and cyan isosurfaces represent the positive and negative spin densities, respectively

Under the biaxial strain of 0~7%, the V_Se_-, V_Se-Se_-, and V_WSe3_-doped monolayer WSe_2_ remain to be non-magnetic as shown in Fig. 5. In contrast, the non-magnetic V_W_-doped monolayer WSe_2_ become magnetic with the magnetic moment of 4 μ_B_ under the biaxial strain larger than 1%. The spin-resolved charge density shown in Fig. 7a indicates that the magnetic moment mainly arises from the W and Se atoms around the vacancies. As shown in Fig. 7b, the magnetic moment of V_W2_-doped monolayer WSe_2_ mainly comes from the Se atoms near the vacancy and little from the W atoms around the vacancy. When the applied strain is larger than 1%, more Se atoms are spin-polarized, resulting in the larger magnetic moment of 4 μ_B_. For V_WSe6_ vacancy defect, we can see that its magnetic moment remains to be 6 μ_B_ under the strain of 0~6% and then decreases to 4 μ_B_ at the strain of 7% as shown in Fig. 6b. Figure 7c demonstrates that its magnetic moments mainly arise from the six W atoms around V_WSe6_. When the applied strain increases to 7%, the nearby Se atoms around the vacancy are more spin-polarized, but the local magnetic moments on the W atoms decrease. Correspondingly, the total magnetic moment of V_WSe6_-doped WSe_2_ decreases to 4 μ_B_ under 7% strain.

## Conclusion

In summary, we studied several vacancy defects for monolayer WSe_2_, including the single Se and W atom vacancies (V_Se_ and V_W_), double Se and W atom vacancies (V_Se-Se_ and V_W2_), big vacancy of one W atom and the nearby three Se atoms on the same layer (V_WSe3_), and vacancy of one W atom and the nearby three pairs of Se atoms (V_WSe6_). The V_Se_-, V_Se-Se_-, V_W_-, and V_WSe3_-doped monolayer WSe_2_ all keep the non-magnetic semiconducting feature as the perfect WSe_2_ monolayer, but with smaller energy gaps due to the impure electronic states locating in the energy gap region, which are attributed from the W d and Se p orbital around the vacancies, while V_W2_ and V_WSe6_ vacancies induced magnetism into monolayer WSe_2_ with magnetic moments of 2 and 6 μ_B_, respectively. Particularly, monolayer WSe_2_ with V_W2_ vacancy converts into half-metal from semiconducting. More importantly, our calculation results show that the external biaxial strain effectively tunes the magnetism and electronic properties of monolayer WSe_2_.

## Abbreviations

2D: Two-dimensional
CVD: Chemical vapor deposition method
DFT: The density functional theory
DOS: The density of states
HSE06: The Heyd–Scuseria–Ernzerh method
PAW: The projector augmented wave method
PBE: The Perdew–Burke–Ernzerhof method
PDOS: The partial density of states
TMDs: Transition metal dichalcogenides
VASP: Vienna Ab initio Simulation Package
